# Long-term field-realistic exposure to a next-generation pesticide, flupyradifurone, impairs honey bee behaviour and survival

**DOI:** 10.1038/s42003-021-02336-2

**Published:** 2021-06-28

**Authors:** Simone Tosi, James C. Nieh, Annely Brandt, Monica Colli, Julie Fourrier, Herve Giffard, Javier Hernández-López, Valeria Malagnini, Geoffrey R. Williams, Noa Simon-Delso

**Affiliations:** 1 Department of Agricultural, Forest, and Food Sciences, University of Torino, Grugliasco (TO), Italy; 2 Division of Biological Sciences, Section of Ecology, Behavior, and Evolution, University of California San Diego, San Diego, CA USA; 3 LLH – Bee Institute, Kirchhain, Germany; 4 Ecotoxicological Unit, Biotecnologie BT S.r.l., Todi, Italy; 5 ITSAP, Institut de l’Abeille, Avignon, France; 6 Testapi SAS, Gennes, France; 7 Inst. f. Zoologie, Karl Franzens Universität, Graz, Austria; 8 Center for Technology Transfer, Edmund Mach Foundation, San Michele all’Adige, Italy; 9 Institute of Bee Health, University of Bern, Bern, Switzerland; 10 Department of Entomology & Plant Pathology, Auburn University, Auburn, AL USA; 11 BeeLife European Beekeeping Coordination, Louvain la Neuve, Belgium

**Keywords:** Toxicology, Entomology, Drug regulation, Agriculture, Animal behaviour

## Abstract

The assessment of pesticide risks to insect pollinators have typically focused on short-term, lethal impacts. The environmental ramifications of many of the world’s most commonly employed pesticides, such as those exhibiting systemic properties that can result in long-lasting exposure to insects, may thus be severely underestimated. Here, seven laboratories from Europe and North America performed a standardised experiment (a ring-test) to study the long-term lethal and sublethal impacts of the relatively recently approved ‘bee safe’ butenolide pesticide flupyradifurone (FPF, active ingredient in Sivanto^®^) on honey bees. The emerging contaminant, FPF, impaired bee survival and behaviour at field-realistic doses (down to 11 ng/bee/day, corresponding to 400 µg/kg) that were up to 101-fold lower than those reported by risk assessments (1110 ng/bee/day), despite an absence of time-reinforced toxicity. Our findings raise concerns about the chronic impact of pesticides on pollinators at a global scale and support a novel methodology for a refined risk assessment.

## Abstract (web summary)

Tosi and colleagues perform a large, multinational experiment on honey bees, encompassing multiple subspecies, to assess the short- and long-term effects of a next-generation pesticide, flupyradifurone, on this key pollinator species. Their findings indicate that this “bee safe” pesticide impairs survival and behaviour at real-world exposure levels.

## Introduction

The increased use of pesticides and fertilisers is a major cause of reduced insect biodiversity^1,2^, and is a concern because insects provide key services to natural and agricultural ecosystems^3^. In particular, reduced bee health is of significant interest and has been linked, in part, to pesticide exposure^4,5^. Pesticide residues can often be found in multiple environmental sources^6,7^, and bees can be exposed while flying, collecting water, and collecting and consuming food resources like nectar, honeydew, or pollen^8–10^.

Among pesticides, the neonicotinoids (Insecticide Resistance Action Committee (IRAC) Group 4A) have received major attention. Used globally since the 1990s^11^, they now face regulatory challenges^12^ due to their demonstrated harmful effects on bees^4,5,13,14^. Pest insects are also developing resistance against them^14^. As a result, new generations of insecticides are entering the market.

Flupyradifurone (FPF, the active ingredient in Sivanto^®^, Bayer CropScience)^15^ was first registered in 2014 in Guatemala and Honduras, then in 2015 in the USA and the EU, and is now available globally^16,17^. FPF is relatively new, and thus few pest species have developed resistance against it^15,17,18^. Like the neonicotinoids, FPF is a systemic insecticide and an agonist of insect nicotinic acetylcholine receptors (IRAC group 4). It can be used to manage a variety of pests on diverse crops (vegetables, potatoes, pome fruits, grapes, citrus, cotton, soybean, coffee, cocoa, hops, and ornamentals) using multiple application methods (spray, drip irrigation, soil treatments, and seed treatments)^17,19^. Although FPF and the neonicotinoids share similar chemical structures^17,20^, FPF is classified by the IRAC as a butenolide insecticide (4D subgroup) due to structural differences involving the pharmacophore^15^. Notably, FPF and all chloropyridinyl neonicotinoids (imidacloprid, thiacloprid, acetamiprid, and nitenpyram)^21^ share a common metabolite, 6-chloronicotinic acid (6-CNA) that is toxic to bees (LC_50_ = 0.01 ng/bee/day)^22^.

Because FPF has been described as relatively ‘bee safe’^23^, with a favourable ecotoxicological safety profile^17^, it can be used on flowering crops while bees are actively foraging. Therefore, bees may be exposed to this emerging contaminant in pollen and nectar or via direct contact. Tosi and Nieh^24^ demonstrated that field-realistic FPF exposures cause sublethal and lethal synergistic effects in honey bees (*Apis mellifera*) when combined with a common fungicide. They showed that FPF reduces bee survival and impairs behaviour (375 ng FPF/bee, oral exposure) and that its toxicity varies across season and bee age: FPF was more toxic to foragers (compared to in-hive bees) and in summer (compared to early spring). Tong et al.^25^ showed that field-realistic 3-day chronic oral exposure to FPF (4000 µg/kg, FPF_daily dose_ = 241 ± 4 ng/bee/day (mean ± Standard Error of the Mean, SEM), corresponding to 1/12 of LD_50,_ 2995 ng/bee^24^) reduced honey bee survival, flight success, thermoregulation, and food consumption in combination with nutritional stress (limited nectar availability) and season (winter vs. summer). Tan et al.^26^ reported that short-term chronic exposure to FPF impaired olfactory learning in larval (33 ng/larvae/day over 6 days) and adult (66 ng/adult bee/day over 3 days) eastern honey bees (*Apis cerana*). Hesselbach and Scheiner^27,28^ found no effects at field-realistic doses, but showed that acute exposure to a high FPF dose (1.2 µg/bee) impaired bee taste, cognition, and motor abilities in *A. mellifera*. A 7-day or 10-day exposure to FPF daily doses of respectively ~120 and ~1000 ng/bee reduced foraging onset and bee survival^29^. Chakrabarti et al.^30^ exposed honey bees to a single acute contact dose of Sivanto^®^ and found reduced survival and increased oxidative stress and onset of cellular apoptosis. Another recent study found that FPF decreased the survival of bees infected with the gut parasite *Nosema ceranae*^31^. Campbell et al.^32^ tested the effects of FPF on field colonies of *A. mellifera* and observed no significant short-term side effects on colony strength. However, bee-collected nectar and pollen sampled from the control fields in their study were also contaminated with FPF, complicating interpretation.

In-hive bees and foragers may be exposed to FPF over extended time periods. After FPF field treatment, bees can ingest FPF-contaminated nectar (detected in residues from bee honey stomachs) for more than two weeks, and FPF-contaminated honey was detected for up to five months^23^. In these initial studies, FPF was only used on target crops to determine bee exposure. However, FPF can be used across different seasons on multiple crops and ornamental plants because of its broad spectrum of pest targets and application methods^17^. Bees may be exposed for longer periods of time due to the use of FPF on multiple sequentially blooming crops or through colonies foraging on different crops treated with FPF. Like the neonicotinoids, FPF can contaminate soil and water for long periods and can be persistent and mobile in the environment^23^.

An official protocol for testing a short, 10-day chronic exposure has only been available since 2017^33–36^. However, it has not been widely adopted in risk assessments^34^, nor has it been used to make final decisions on chemical safety (i.e. FPF) because of a crucial lack of harmonised methodologies and practices^37^. Since bees can be chronically exposed to pesticides for longer periods^8,10^, and because such prolonged exposure can increase toxicity^22,38–41^, we assessed the long-term effects of FPF, including its Time-Reinforced Toxicity (TRT, also called time-dependent or time-cumulative toxicity)^41^. We employed a time-to-death approach in which we monitored individuals until at least 50% of controls died. Seven laboratories located in six countries in Europe and North America participated using the same protocol and exposed multiple subspecies of honey bees to a range of field-realistic FPF daily doses to obtain lethal and sublethal information on their health and risk assessment.

Our work coordinates research on pesticides and risk assessment across multiple countries and continents, a model for future studies. We propose innovative assessments of pesticide toxicity in bees and other insects, revealing long-term lethal and sublethal effects of FPF, a pesticide considered “bee safe”. We demonstrate that long-term exposure to low, field-realistic levels of FPF reduces bee survival and food consumption, mainly over longer periods, and increases bee abnormal behaviours over the short term. We conclude that long-term and sublethal effects should be routinely investigated by research and risk assessments to safeguard bees and our environment.

## Results

The validity criteria stated in the official guidelines^36^ were met by all participating laboratories. After 10 days, the mortality of the negative control (pure 50% sucrose solution) was ≤ 15%, and the mortality of the positive control (DIM, 1000 µg/kg) was ≥ 50%. These results used the standard thresholds reported in official guidelines for ecotoxicological testing of bees^36^, tested the sensitivity of the bees, and demonstrated the reliability and reproducibility of our protocol.

A ring test (also called a proficiency test) is a standard trial that requires multiple laboratories to follow the same protocol. A ring test is typically used for quality assurance through inter-laboratory comparison of test performances. The laboratories provided satisfactory results within our ring-test framework (z-score < 2, Supplementary Table 1). In total, we tested 150 bee groups (cages) for a total of 2879 bees from 21 colonies. The overall duration of the trial was 31 ± 5 days (mean ± Standard Error of the Mean, SEM), and the time until the death (Lethal Time, LT) of 50% (LT_50_) of the control treatment was 25 ± 2 days.

### Field-realistic FPF exposure decreased survival

**Fig. 1:**
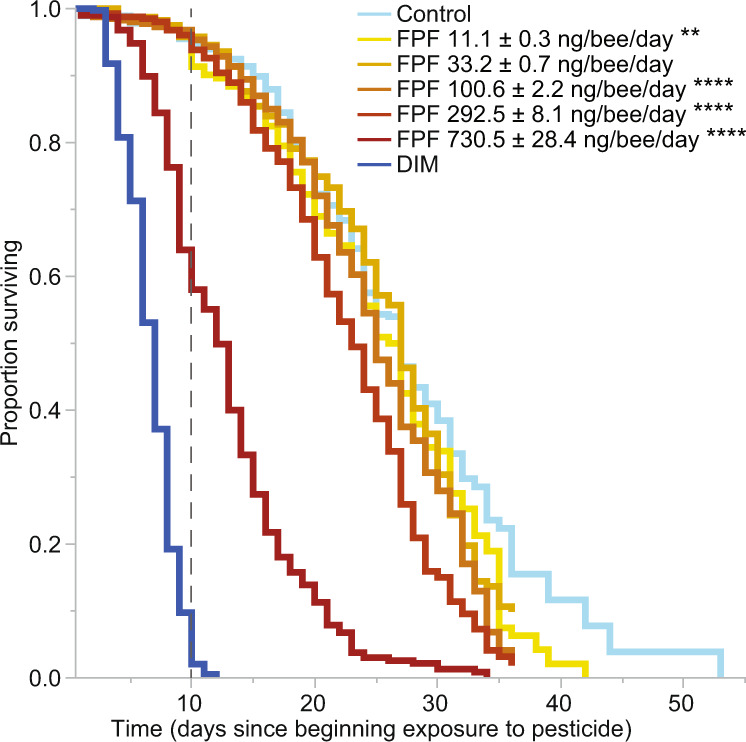
Effects of flupyradifurone (FPF) and dimethoate (DIM) on bee survival. We have indicated the end of the 10-day standard test period^36^ with a dashed line to facilitate comparison between the standard chronic risk assessment trial (10-day) and our longer term experiment. Only the highest FPF treatment (730.5 ± 28.4 ng/bee/day, mean ± Standard Error of the Mean, SEM) caused a significant effect before 10 days (*p* < 0.0001), while the longer exposure allowed us to determine the significant effects of lower doses (11.1 ± 0.3 ng FPF/bee/day (mean ± SEM): *p* < 0.01; 100.6 ± 2.2: *p* < 0.001; 292.5 ± 8.1; *p* < 0.0001; 730.5 ± 28.4: *p* < 0.0001; Supplementary Table 3). In the legend, asterisks indicate the significant differences between the respective FPF dose and control in the long term (Kaplan-Meier; *n* = 2494; **p = 0.01, ****p = 0.0001). Different colours indicate different treatments. Further survival details are in Supplementary Tables 2–4.

The active ingredient FPF significantly reduced adult worker bee survival (survival was monitored in bees from their emergence up to 31 ± 5 days, *p* < 0.0001, Fit Proportional Hazards, Fig. 1, Supplementary Tables 2–4, *n* = 2494). Bee survival was significantly impaired by all but one FPF daily dose when compared to the control treatment (Fig. 1, Supplementary Tables 3–4). The lowest FPF treatments mainly decreased survival over longer periods of exposure (Supplementary Table 4). FPF significantly reduced bee survival over the first 10 days (*p* < 0.0001, Fit Proportional Hazards, Fig. 1, Supplementary Tables 2–4), but only when the bees ingested the highest FPF daily dose of 731 ± 28 (mean ± SEM) ng/bee/day (measured from consumption, *p* < 0.0001, Kaplan–Meier, Dunn–Sidak corrected, Supplementary Table 3).

There was no main effect of bee subspecies (*A. m. carnica*, *A. m. ligustica*, or *A. m. buckfast*) on bee survival (Kaplan–Meier, *p* = 0.18, DF = 2, χ^2^ = 3.44).

### Field-realistic FPF exposure decreased food consumption

**Fig. 2:**
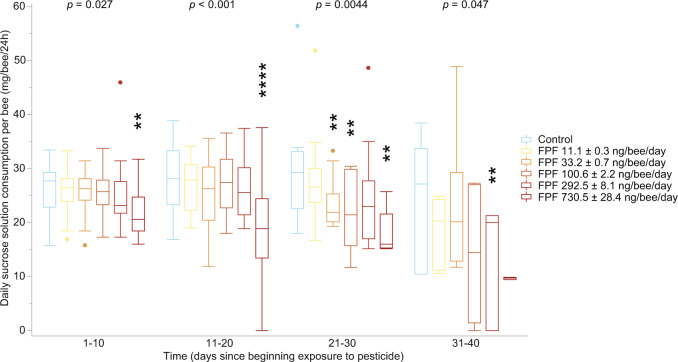
Effects of flupyradifurone (FPF) on daily sucrose solution consumption per bee. We assessed our results in time blocks of 10 days to facilitate comparison between the standard 10-day risk assessment chronic test and longer term exposures^36^. We report the significance of the main effect of FPF treatment above each time period (Supplementary Table 5). Asterisks indicate significant differences between the respective FPF dose (reported in ng/bee/day, mean ± Standard Error of the Mean, SEM) and control (GLM^DS^; *n* = 111; **p < 0.01, ****p < 0.0001; Table 10). The box-plot reports the maximum (excluding outliers), the percentiles (75th, 50th, and 25th), the minimum (excluding outliers), and the outliers. Additional details, including effect sizes, are in Supplementary Tables 5–7.

FPF significantly reduced bee food consumption at all time frames (main effect comparing the overall effect of FPF across time periods; *p* ≤ 0.047, GLM, Fig. 2, Supplementary Table 5, *n* = 111). This effect was significant at several FPF daily dose treatments (GLM, Least-Square Means contrast test, Dunn–Sidak corrected, Fig. 2, Supplementary Tables 6, 7). Although we corrected our food consumption data with the number of living bees and the daily evaporation rate, the increased bee mortality in the period between 31–40 days (i.e., 32 cages with bees alive after 31 days) led to greater food consumption variability (SEM = 2), potentially contributing to a non-significant result (Fig. 2).

Our work also provides critical information on the amount of sucrose solution and FPF consumed by bees over long periods and provides a baseline for honey bee consumption over most of a bee’s lifespan (Supplementary Tables 8, 9).

### Field-realistic FPF exposure increased abnormal behaviours

**Fig. 3:**
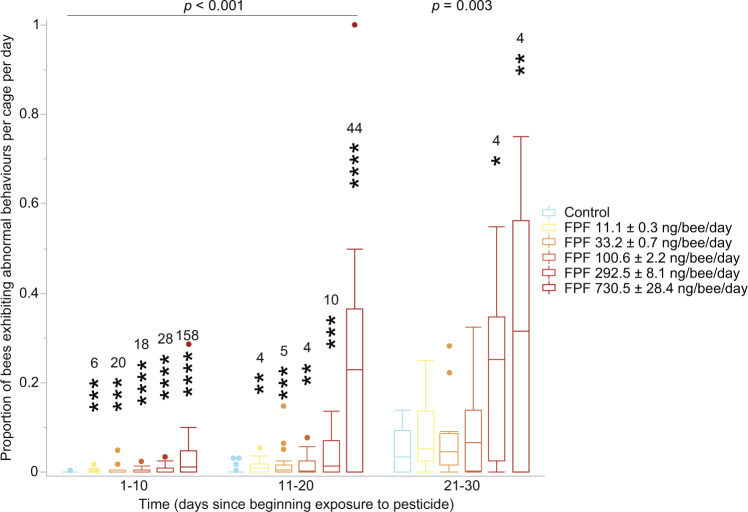
Effects of flupyradifurone (FPF) on the daily proportion of living bees exhibiting at least one abnormal behaviour per cage. We assessed the results in time blocks of 10 days to allow comparison between the standard 10-day risk assessment chronic test and longer term exposures^36^. We report the significance of the main effect of FPF treatment above each time period (Supplementary Table 10). Asterisks above the bars indicate significant differences between the respective FPF dose (reported in ng/bee/day, mean ± Standard Error of the Mean, SEM) and the controls (GLM^DS^; *n* = 111; *p = 0.05, **p = 0.01, ***p = 0.001, ****p = 0.0001; Supplementary Table 11). We report the effect sizes measured in fold-increase above each significant effect to facilitate interpretation given the low absolute values of the control treatment and the lower FPF doses (Supplementary Table 12). The box-plot reports the maximum (excluding outliers), the percentiles (75th, 50th, and 25th), the minimum (excluding outliers), and the outliers. Additional details are in Supplementary Tables 10, 12.

FPF significantly increased the frequency of bees exhibiting abnormal behaviours (e.g. motion coordination deficits, hyperactivity, and apathy) between 1–30 days after treatment (*p* < 0.003, GLM, *n* = 111) at all FPF daily dose treatments (GLM, Least-Square Means contrast test, Dunn–Sidak corrected, Fig. 3, Supplementary Tables 10–12). Details and definitions on the types of abnormal behaviours exhibited by bees after pesticide exposure are based on recently published research investigating risk assessment methods^24^. We also used a video and its text description to train researchers to identify common abnormal behaviours observed after oral pesticide consumption in bees^24^. Additional details are in the main text of this manuscript.

### FPF does not cause time-reinforced toxicity

**Fig. 4:**
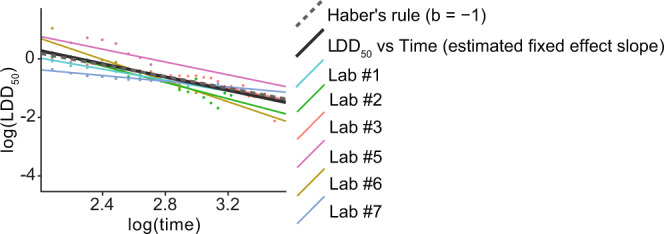
Flupyradifurone (FPF) time-reinforced toxicity assessment. There is no evidence of time-reinforced toxicity of FPF in honey bees, as its toxicity (estimated global regression of the Lethal Daily Dose causing 50% mortality, LDD_50_; black solid line) over time (days) was not significantly different from Haber’s rule (random slope mixed model; dashed grey line with a slope of −1). We display separate regression lines, using different colours, for each laboratory. Further details are available in the Supplementary Methods and Results.

We did not find evidence of time-reinforced toxicity of FPF in honey bees. Since the slope of the regression between the log(LDD_50_) and log(Time) was not significantly different from −1 (estimated slope = −1.13 with 95% bootstrap CI [−1.49, −0.7]), we were not able to reject Haber’s rule (Fig. 4, see Supplementary Methods and Results for more details). Haber’s rule assumes that a toxic effect (i.e., mortality) of a chemical is described by a constant linear relationship between its level and its duration of exposure (i.e., time to death)^42^. According to this rule, toxicity is not cumulative when reducing the dose increases the time to achieve the same level of toxicity by an equal proportion (yielding a −1 slope for the log—log regression between toxicity and time). Time-reinforced toxicity would occur if the dose—response relationship violates Haber’s rule. Incomplete detoxification could cause bioaccumulation and consequently reinforce toxicity over time (further details in the SI methods, results, Supplementary Fig. 1, Supplementary Table 13).

## Discussion

By performing a large, multinational experiment, we demonstrated that chronic consumption of low, field-realistic levels of flupyradifurone (FPF, Sivanto^®^) has lethal and sublethal effects on honey bees. We provide the first evidence that FPF reduces bee survival (444 µg/kg, corresponding to 11 ng/bee/day, 273-fold lower than its LD_50_ of 2995 ng/bee^24^) and food consumption (1333 µg/kg, corresponding to 33 ng/bee/day) mainly over longer periods (i.e., after 20 days), and increases bee abnormal behaviours (400 µg/kg) in the short term. Importantly, we demonstrate that current laboratory risk assessments of pesticides most likely underestimate their impact because the 10-day observation period is too short and too focused on lethal impacts.

Over time, the adverse effects of FPF on survival and food consumption became stronger and more evident, even at lower doses. These effects would not be measured with the standard 10-day trials recommended for official risk assessments^23,36,37^. This is likely true for multiple other pesticides because their registration processes require equal or more lenient testing than FPF registration. Although the risk assessment of FPF included a facultative 10-day chronic trial, the results were at times disregarded or only considered as additional information due to procedural inadequacies, including a lack of harmonised methodology^23,37,43^. Our experiment captured sublethal effects at a 101-fold lower level as compared to the 10-day chronic results produced during the FPF risk assessment registration trials (Low Observed Adverse Effect Concentration (LOAEC) = 41000 µg/kg, corresponding to 1110 ng/bee/day)^44^. We also showed that FPF causes impairments at 91-fold (on survival) and 11-fold (on behaviour) lower doses as compared to a recent work testing 10-day exposure (~1000 and ~100 ng/bee/day, respectively)^29^.

Our results accord with prior research demonstrating that FPF, like neonicotinoids^4,45–47^, can increase abnormal behaviour even in the short term^24,25^. These other studies were performed under different experimental conditions (i.e., did not test newly emerged bees or use our specifically adapted exposure and bee rearing methodologies), and thus their specific dose effects are not comparable with our multinational chronic experiment. However, this prior research provides valuable information on the timing and types of adverse FPF effects on bees. Abnormal behaviours that alter bee efficiency and fitness temporarily can have multiple impacts. For example, a bee that is intoxicated in the field, even for a short time, might have altered flight^45^, locomotion^46^, and orientation^48^ abilities, or any of these deficits, and might thus be unable to return to its colony^47^. A reduction of bee foraging efficiency may ultimately have a broader effect by weakening the pollination services provided^5^. Further work is recommended to determine the colony level impacts of individual behavioural deficits^33^.

We provide the first evidence of no time-reinforced toxicity of a next-generation butenolide insecticide, allowing comparisons with the impact of previous common pesticides suspected of playing a significant role in bee health declines like the neonicotinoids^41,49–51^ and fipronil^52^. Bees may be efficient at clearing or detoxifying FPF since we did not find evidence of accumulating FPF effects (Fig. 4). Actual measures of FPF levels in bees over time are needed to confirm this hypothesis. However, bees under certain treatments ingested, in the long term, a cumulative dose of FPF that should reach (or even be greater than) the LD_50_, but their respective mortality was not similarly increased. We hypothesise that FPF may be ~700-fold less toxic than N-nitroguanidine neonicotinoids (i.e., thiamethoxam, clothianidin, imidacloprid, based on their LD_50_) because bees can detoxify this molecule at greater efficacy and reduce its bioaccumulation, as has been shown for N-cyanoamidine neonicotinoids^42,53^. In fact, N-nitroguanidine neonicotinoids toxicity is typically reinforced over time due to accumulation, while N-cyanoamidine neonicotinoids and FPF toxicities seem to remain more stable over time^41,42^. Interestingly, the agricultural pest silverleaf whitefly (*Bemisia tabaci*) cytochrome P450 CYP6CM1 metabolises N-nitroguanidine neonicotinoids (i.e., imidacloprid: 23% degradation after 4 h) but not FPF^17^. Furthermore, Tosi and Nieh^24^ demonstrated that FPF toxicity is synergistically amplified (Model Deviation Ratio = 4, meaning that the magnitude of the synergistic effect is four times greater than additivity) by non-lethal levels of a common fungicide (propiconazole; Sterol Biosynthesis Inhibitor, SBI) that inhibits cytochrome P450 detoxification. This strong synergistic effect occurs when the ability of bees to detoxify FPF is impaired, and this supports the hypothesis that honey bees are efficient at detoxifying FPF. These results pose a major challenge because current risk assessments only evaluate the risk of one pesticide at the time^54–56^. We recommend testing for potential synergies of pesticides that have a greater probability to cause harmful interaction effects based on their chemical characteristics (i.e., mode of action)^24^ and their likelihood of co-exposure in the real world.

Our findings suggest hormesis, a non-monotonic biological response that can typically cause apparent positive effects (i.e., stimulation) at low levels and adverse effects (i.e., inhibition) at high levels. Hormesis is a relatively common effect of pesticides, but is complex to investigate and can vary depending on timing and exposure levels^57–59^. In our case, survival was significantly reduced in the long term by all daily doses except an intermediate one (33 ng/bee/day). This result is in line with a previous study that showed that FPF synergistic effects were more evident at lower and higher doses, but not at intermediate ones^24^. Neonicotinoids also show similar effects in bees^60^. In general, the reasons underlying such non-linear variation in toxicity should be further explored.

Our multinational results provide a baseline for honey bee survival, consumption, and food evaporation rate in the laboratory over most of the bees’ lifespan (Supplementary Tables 1, 4, 8, 9, 16). Thus, our findings allow developing validity criteria for longer term studies. We propose using Lethal Time (LT) outcomes to guarantee a minimum and maximum LT for, respectively, the negative and the positive control treatments. Measuring the LT of different percentages of the population (25%: LT_25_, 50%: LT_50_, and 75%: LT_75_) would allow greater accuracy. Based on our survival results (LT_25, control_ = 20 days, LT_50, control_ = 27 days, LT_75, control_ = 34 days), we suggest an LT_25_ of 15 days, an LT_50_ of 20 days, and an LT_75_ of 25 days as minimum acceptable thresholds for honey bee negative controls in the long term. The validity criteria for positive controls should use dose levels that are appropriate to the longer duration of the test. Our work used the standard positive control levels suggested by the Organisation for Economic Co-operation and Development (OECD)^36^, which typically result in reaching the LT_50_ before 10 days. We therefore suggest using lower positive control levels (i.e., 0.1 mg/kg, a tenth of that suggested in OECD) to ensure that sufficient bees survive to measure longer term mortality.

To better protect insect pollinators and our environment, we recommend implementing truly long-term tests to assess sublethal, synergistic, and time-reinforced interactions in pesticide research and risk assessment. We also suggest a more accurate and thorough assessment of abnormal behaviours, possibly made more feasible for mass assessments by publicly available behavioural descriptions and video analyses adapted for standard risk assessments^24^. Our ring-tested time-to-death approach, performed in seven laboratories (six countries, two continents), showed the validity of our harmonised procedure on a broad spatial scale. These methods could thus be adapted to assess pesticide lethal and sublethal effects in insect pollinators and other non-target organisms, allowing a greater understanding of pesticide risks and improving environmental protection.

## Methods

This multinational study was conducted during the 2016 and 2017 active beekeeping seasons. Seven laboratories based in six different countries participated following a common protocol—a ring test (Supplementary Table 1). A ring test is a standard trial that involves multiple laboratories following the same protocol, and allows evaluating the performance of testing laboratories^61^. We used a total of 21 *A. mellifera* honey bee colonies (three per laboratory) that were considered healthy based upon standard inspection techniques^62,63^. The *A. mellifera* subspecies are recorded in Supplementary Table 1.

### Refinement of official guidelines

We followed international guidelines for testing chronic oral toxicity of chemicals on honey bees^36^. However, we introduced multiple improvements. We (1) tested a higher number of colonies per treatment (three colonies, not just one), (2) tested a higher number of bees per replicate (20 bees vs. 10), and (3) used prolonged exposures (at least up to the LT_50_ of the control treatments instead of only 10 days, Supplementary Table 14). Instead of a fixed trial duration (10 days), we used a time-to-death approach so that the experiment continued until at least 50% of the control bees died (31 ± 5 days, mean ± SE).

### Flupyradifurone (Sivanto^®^) and dimethoate concentration and doses

Feeding solutions with 50% (w/v) sucrose were either pure (negative control treatment, only sucrose solution) or contained DIM (positive control, 1 mg/kg)^36^ or FPF. The insecticide DIM is typically used as positive control to evaluate the exposure of the tested toxin^64^. We tested five daily doses of FPF (11.1 ± 0.3, 33.2 ± 0.7, 100.6 ± 2.2, 292.5 ± 8.1, and 730.5 ± 28.4 ng/bee/day, mean ± SEM) that resulted from feeding bees different FPF concentrations (444, 1333, 4000, 12000, and 36000 µg FPF/kg sucrose solution) (Supplementary Table 8). The different daily intake of test solutions caused, expectedly, a limited variability of daily dose FPF intakes, here reported as SEM (Supplementary Table 8). These FPF levels were chosen to cover a field-realistic range (including very low doses) based on available data, preliminary (range finding) tests, and official ecotoxicological guidelines^33,36,64^. We used a geometric series with a common ratio factor of three starting from the middle concentration^24^ based upon exposure data (see below and Supplementary Methods).

Our FPF daily doses reflect field-realistic exposures because FPF can be found in colonies up to approximately five months after exposure^23^, and honey bees can ingest daily doses of FPF that are higher in the field as compared to those we administered (see above, Supplementary Methods, and previously published assessments^23–25^).

Honey bees can ingest FPF concentrations up to 4300 µg FPF/kg in nectar and 21000 µg/kg in pollen when foraging oilseed rape crops. We further estimated the actual dose of FPF ingested to measure exposure more accurately, following European Food Safety Authority (EFSA) and US Environmental Protection Agency (EPA) methods (Supplementary Methods). To do so, we used FPF residues in nectar (4300 µg/kg) and pollen (21000 µg/kg) of oilseed rape^23^ and standard consumption data^33^. We used oilseed rape because this is a standard for research and exposure assessment^33^. In terms of dosages, foragers collecting nectar in a field previously sprayed with FPF can be exposed to 5504 ng FPF/bee per foraging day (worst-case scenario)^33^. Unlike foragers, nurses ingest less nectar and more pollen, leading to an exposure of 2402 ng FPF/bee/day (worst-case scenario)^33^. Further details are reported in the Supplementary Methods.

The highest FPF daily dose we tested was ~4 times lower than the LD_50_ of FPF. We calculated the LD_50_ of FPF (2995 ng/bee)^24^ at the beginning of this experiment with honey bees collected from one of our study apiaries. This highlights the variability of FPF lethal toxicity, since previous studies found an LD_50_ of 1200 ng/bee^23^. Such LD_50_ discrepancies have been observed in the neonicotinoids, systemic insecticides that are also agonists of insects nicotinic acetylcholine receptors (nAChRs, IRAC Group 4D)^65,66^. Because LD_50_ variability depends on specific experimental conditions^66,67^, we considered the value calculated in Tosi and Nieh^24^ to be a more relevant reference point as compared to the LD_50_ calculated years earlier under different conditions^23^. We used 11 ng/bee/day as the lowest FPF daily dose because it was much lower than the doses tested in published studies that used a dose–response design (i.e., 272-fold lower than its LD_50_, and 101-fold lower than the lowest observed adverse effect level identified by the US EPA)^23,29,44^.

Stock solutions of FPF (CAS #: 951659-40-8, Purity: 99.9%, PESTANAL analytical standard, Sigma-Aldrich Laborchemikalien GmbH) and dimethoate (CAS #: 60-51-5, EC #: 015-051-00-4, Purity: 99.5%, PESTANAL analytical standard, Sigma-Aldrich Laborchemikalien GmbH) were prepared with deionized water and stored at 4 °C^23^. Pesticides are typically applied as mixtures, but we did not test the Sivanto^®^ formulation because official ecotoxicology testing guidelines require using the active ingredient to avoid confounding factors (i.e., synergies, variability in the formulation composition, and impurities that are more likely in formulations as compared to active ingredients)^33,36^. We did not use acetone because both compounds are soluble in water at the stock concentrations used (FPF: 3,200 mg/L, dimethoate: 39,800 mg/L). The final feeding solutions were prepared by diluting the stock solution with 50% (w/v) aqueous sugar solution. Feeding solutions were provided *ad libitum* to the bees at the beginning of trial and renewed each 24 ± 2 h. These dilutions were prepared at least once every 4 days, were tightly wrapped with aluminium foil to prevent light degradation and stored at 6 ± 2 °C^23^. Feeding solutions had no sign of precipitation at any time.

### Honey bee preparation

We collected brood frames with capped cells within one day of adult emergence from each experimental colony. Frames were maintained at 34.5 ± 1 °C and 50–80% RH without nurse bees (Day −2)^68^. The next day, we moved the newly emerged bees from the combs to test cages, 20 bees per cage and each cage only housed bees from the same colony (Supplementary Table 1). We did not use anaesthesia, and followed standard methodologies to feed the bees and provide the experimental treatments^36,64,67^. Each test cage received one only feeder (a plastic syringe with the tip cut off to facilitate solution flow^68^) which contained one experimental treatment in 50% sucrose solutions (w/v). The feed solution was provided ad libitum, and the control treatment corresponded to pure sucrose solution only. We exchanged these syringes for fresh ones (belonging to the same treatment group) each day (24 ± 2 h).

Cages were maintained in darkness at 33 ± 2 °C, and 50–70% RH. We maintained the bees in the cages for one day (from Day −1 to Day 0) before the beginning of the trial (Day 0) to allow acclimatisation to test conditions. All experiments began with newly emerged bees of maximum 2 days of age, and continued until at least the LT_50_ (i.e., 50% mortality of the control treatment was reached). Some laboratories were able to continue the assessments beyond this 50% mortality point (Supplementary Table 1).

### Observations

We recorded abnormal behaviours, survival, and food consumption each 24 h. Every day, we weighed the sucrose solution feeders. Separately, each laboratory used three cages maintained in identical conditions, but without bees, to measure the average mass loss due to evaporation of sugar solutions from the syringes and accounted for this evaporative mass loss in the calculations (average evaporation rate = 1.5%, maximum evaporation rate = 2.3%, Supplementary Table 15). A bee was considered dead when it was immobile and did not react to stimulation^67^. We calculated the mean daily solution consumption (g of solution) per living bee. This daily solution consumption calculation was based on the weight of sugar solution consumed by each cage daily, corrected by the number of alive bees per cage per day and by the evaporation rate per laboratory per day. In total, we tested food consumption and abnormal behaviours of 111 groups (cages) for a total of 2220 bees. Behavioural abnormalities were quantified according to the categories described in the standard OECD guidelines^36^, and further refined using recently published behavioural protocols and videos^24^. Bee behaviours were observed in person at the time of the daily assessment, following established methods^24^. These abnormalities were grouped into five categories (motion coordination deficits, hyperactivity, apathy, curved-down abdomen, and moribund) and are based upon previously published descriptions^24^. We used a video to describe with text and images the common abnormal behaviours observed in ecotoxicological trials^24^ for training and reference purposes. This video is recommended for future behavioural assessments. To facilitate current and future behavioural assessment across multiple continents and laboratories, we simplified our scoring of abnormal behavior to simply score its presence or absence per bee each 24 h.

### Statistics and reproducibility

We used Fit Proportional Hazards models to test the effect of FPF treatment, colony, and laboratory on bee survival (Fig. 1, Supplementary Table 2, *n* = 2494 bees)^69^. Colony and laboratory were not considered as main factors. Significant effects were further analysed with Kaplan–Meier survival analyses (Log-Rank Chi-square values) following visual data inspection (Supplementary Table 3)^70^. We calculated the LT_50_, LT_25_, and LT_75_ at each FPF treatment tested, and across short-term (10 days^36^) or long-term (31 ± 5 days, our complete experiment) exposures (Supplementary Table 4). Per each period (10 vs 31 ± 5 days), we also reported the Risk Ratios (RR) calculated between each FPF dose and the control treatment. The RR value measures how the risk of death increases in pesticide-exposed bees as compared to controls, and the respective *P*-values were calculated with chi-square statistics (Supplementary Table 3). We separately used the Kaplan–Meier survival analyses to test the effect of bee subspecies on survival.

We used Generalized Linear Models (GLMs) to test the effect of FPF treatment, colony, and laboratory on food consumption (sucrose solution, Gaussian distribution, reciprocal link, Fig. 2, Supplementary Table 4, n = 111) and frequency of bees exhibiting abnormal behaviour (exponential distribution, reciprocal link, Fig. 3, Supplementary Table 10, n = 111) separately for each 10-day interval^71^. We tested 10-day time ranges to compare the standard 10-day chronic test^36^ and longer term exposures. To do so, we averaged the values of food consumption and frequency of bees exhibiting abnormal behaviour for each 10-day range (1–10, 11–20, 21–30, 31–40) by cage. Colony and laboratory were not considered as main factors. We confirmed the suitability of GLM distributions and links with data distribution (i.e., abnormal behaviours data were skewed with non-negative continuous responses) and residual analyses^72^. Binomial models should be typically used for proportions but are not appropriate for our case since we did not compute a ratio of two integers numbers but an average of proportions^72^. Based upon visual data inspection, effects were further analysed with post-hoc Least-Square Means contrast tests (Supplementary Tables 6, 11). We report the effect size measures in Supplementary Tables 7, 12. We used the Dunn–Sidak method to correct for multiple comparisons (*k* = 5, adjusted α = 0.01021, superscript ‘DS’ indicates that the statistical tests passed this correction).

To test for FPF time-reinforced toxicity, we first computed the Lethal Daily Dose causing 50% of mortality (LDD_50_) for each time point (one LDD_50_ per day) using a logistic model (R package drc^73^). Then we fitted a regression line of log(LDD_50_) vs log(Time) using a Linear Mixed Model with the laboratory as a random effect (R package lme4^74^). We computed the confidence interval of the mixed model slope (using 250 parametric bootstrap simulations) to test if it was significantly different from −1 (Haber’s rule). Detailed data analyses (including the R code) are provided in the Supplementary Methods.

We used the z-test as quantitative criteria to evaluate the performance of the different laboratories within the ring test^61,75^. The z-score is calculated as reported in Eq. 1:

1 $$z-{{\rm{score}}}=\frac{{\rm{|}}x-\bar{x}{\rm{|}}}{\sigma }$$

where $x$ is the LT_50_ of a laboratory, $\bar{x}$ is the mean of the LT_50_ of all laboratories, and $\sigma$ is the standard deviation of all laboratories. For LT_50_, we used the combined value including all dose treatments. To assess inter-laboratory performance, the z-score was interpreted using the following common classification: | z | ≤ 2: satisfactory result; 2 < | z | ≤ 3: questionable result; and | z | > 3: unsatisfactory result. Among the seven laboratories that participated in the experiment (Supplementary Table 1), one (Lab #4) was excluded from the sublethal (food consumption, abnormal behaviours) assessments because 15 bees per cage instead of 20 were used. Data from this laboratory were maintained in the Fit Proportional Hazards survival analysis because survival observations (always corrected by number of bees per cage) are more robust to variation of number of bees per cage than sublethal ones (the LT50 of Lab #4 was within the range of other laboratories, and significance was similarly maintained when data from this laboratory were included or excluded) allowing a greater sample size. The LT_50_ of Lab #5 was not met by day 17, when their data were censored (Supplementary Table 1, technical issues). Thus, Lab #5 could not be used in the inter-laboratory assessment as it required reaching the LT_50_. To summarise, seven laboratories contributed data to the survival analysis, and six laboratories contributed data for the sublethal, inter-laboratory, and time-reinforced toxicity assessments.

Our statistical models were run with JMP v14.0.0 (SAS Statistical Software) and R Studio^76^ software. We used residuals analysis to confirm that our data met parametric assumptions. We report mean ± 1 SEM and an alpha value of 0.05. We applied the Dunn–Sidak method^77^ to correct for multiple comparisons when appropriate and indicated with ^DS^ the corrected statistical tests. We applied stepwise model simplification, building models with all interactions, and then removing them if they were not significant.

### Reporting summary

Further information on research design is available in the Nature Research Reporting Summary linked to this article.

## Supplementary information

Supplementary Information

Reporting Summary

## Data Availability

The survival and behavioural data used are available in the open access data repository Figshare (10.6084/m9.figshare.14269706).

## Code availability

The code used is available in the open access data repository Figshare (10.6084/m9.figshare.14269706).
