# Supplementary material for: Long-term field-realistic exposure to a next-generation pesticide, flupyradifurone, impairs honey bee behaviour and survival
Source: Commun Biol. 2021 Jun 28;4:805. doi: 10.1038/s42003-021-02336-2 (PMC8238954; doi:10.1038/s42003-021-02336-2)
Supplement: Supplementary file 1 — Supplementary Information [file 42003_2021_2336_MOESM1_ESM.pdf]

Supplementary Information for

Long-term field-realistic exposure to a next-generation pesticide, flupyradifurone, impairs honey bee behaviour and survival

Simone Tosi*, James C. Nieh, Annely Brandt, Monica Colli, Julie Fourrier, Herve Giffard, Javier Hernández-López, Valeria Malagnini, Geoffrey R. Williams, Noa Simon-Delso

Supplementary Methods

Flupyradifurone (active ingredient of Sivanto®) concentration and doses

FPF is a relatively recently introduced pesticide, and limited environmental contamination data are available[1,2]. Concentrations of 4300 µg/kg and 4108 µg/kg of FPF were found in the honey stomach of foragers collecting nectar from oilseed rape fields treated with FPF in France and Northern Germany[2]. Pollen collected by bees foraging on oilseed rape fields contained 21000 µg/kg of FPF[2]. In other crops, bees can be exposed to FPF at even higher concentrations for longer periods. Bees have been shown to ingest FPF when collecting cotton nectar (22000 µg/kg), apple pollen (39000 µg/kg), or blueberry pollen (68000 µg/kg)[2].

We simulated a scenario in which bees were foraging on oilseed rape crops, and used FPF residues in nectar (4300 µg/kg) and pollen (21000 µg/kg) of oilseed rape. We used oilseed rape crops as reference as they are commonly used for exposure assessment. Our foragers' intake calculation (5504 ng FPF/bee) was based on EFSA[3] and used the highest field-realistic empirical FPF concentration found in the honey stomachs of bees that were collecting nectar from oilseed rape crops (4300 µg/kg)[2] given the average sugar concentration of oilseed rape nectar (10% w/w[4,5]). This sucrose solution was only used to estimate realistic consumption of FPF by bees foraging in oilseed crops[6]. Our nurses' intake calculation (2402 ng FPF/bee/day) was based on EFSA guidelines[3] and considered intake of FPF contaminated pollen using the highest field-realistic FPF concentration in oilseed rape pollen (21000 µg/kg[2]).

According to other calculations[7], the refined Estimated Environmental Concentration (EEC) of FPF is respectively 970 ng/bee and 1256 ng/bee for nurses and foragers when colonies forage in oilseed rape crops[2]. When bees forage on cotton nectar, the refined EEC for workers reaches 6370 ng FPF/bee[2]. Thus, even our highest FPF daily dose of 731 ± 28 ng FPF/bee (mean ± SE) is field-realistic (though worst-case) because nurses and forager bees can consume higher daily doses of FPF when exposed to nectar and/or pollen from oilseed crops. Bees could be exposed to higher doses on other crops.

After application, FPF has been found in nectar and honey stored in wax combs for up to five months, and in nectar collected by foragers for more than two weeks (winter oilseed rape fields[2]). Studies that have measured FPF in-hive contamination showed similar concentrations to those found in food collected by bees outside the hive (up to ~4000 µg/kg), confirming the validity of our approach[2]. Additional monitoring is needed to identify and clarify the duration and level of FPF contamination in the field and in honey bee food under diverse conditions.

Time-reinforced toxicity: statistics

According to Haber's rule, if there is no time-reinforced toxicity, the toxicity of the chemical does not increase over time. For example, if the dose is halved, the time needed to reach the same level of toxicity is doubled (yielding a −1 slope for the log—log regression between toxicity and time).

Two main traditional ways are typically used to assess if the concentration ~ time relationship follows Haber's rule:

1. fit a model of log(Concentration) vs log(LTx)
2. fit a model of log(LCx) vs log(Time) or log(LDDx) vs log(Time)

where LTx, LCx, and LDDx respectively correspond to the Lethal Time, Lethal Concentration, and Lethal Daily Dose to reach x% mortality. If the toxicity follows Haber's rule, the slope of these models should be approximate to −1. If there is time-reinforced toxicity, the slope should be lower than −1. Other endpoints

than 50% might be used too with a general notation: LTx, LCx or LDDx where “x” stands for any level of mortality.

Here, for each of these three approaches and lethal level (from 10% to 90%, using 10% incremental steps), we fit one mixed model regression (random slope model, log—log relationship between concentration and time) for the whole dataset using laboratory as random effect. This mixed model approach provides better estimates by using the whole dataset at once. With this global random slope mixed model, we obtain two types of information:

1. An estimate of a separate slope for each laboratory called “BLUP” (Best Linear Unbiased Predictor) that takes into account the quality of the data in each laboratory (for example a laboratory with fewer points will have an estimate closer to the global average slope);
2. A “fixed effect” slope that is a global average estimate of the slopes of each laboratory.

To compute the confidence interval of the fixed effect global slope, we used 250 parametric bootstrap simulations.

The mixed models were computed with the lme4 package[8] and the LTx, LCx and LDDx values were computed with the drc R package[9] using a logistic dose—response curve.

We provide the statistical details of this analysis, including the R script in this Supplementary Information (SI; SI Methods, SI Results, Fig. S1, Supplementary Table 15) and via the public repository Figshare.

Supplementary Results

Time-reinforced toxicity of FPF in bees

Our time-reinforced results show that none of the slopes is significantly different than −1 (Haber’s rule) (Fig. 4 and Fig. S1, Supplementary Table 15) whatever the methodological approach or the mortality level considered. Thus, the concentration/dose vs time relationship follows Haber’s rule. For the LCx vs Time and LDDx vs time models (Fig. S1), the slope estimate is approximate to −1. Although the slope estimate of the Concentration vs LTx models is often much smaller (around −2, Fig. S1), the precision of the estimates is much lower (larger confidence intervals) and the models were also more unstable, possibly because this regression is based on five points (five concentrations) only, while the other regressions have typically one point per day.

Supplementary Information Figures and Tables

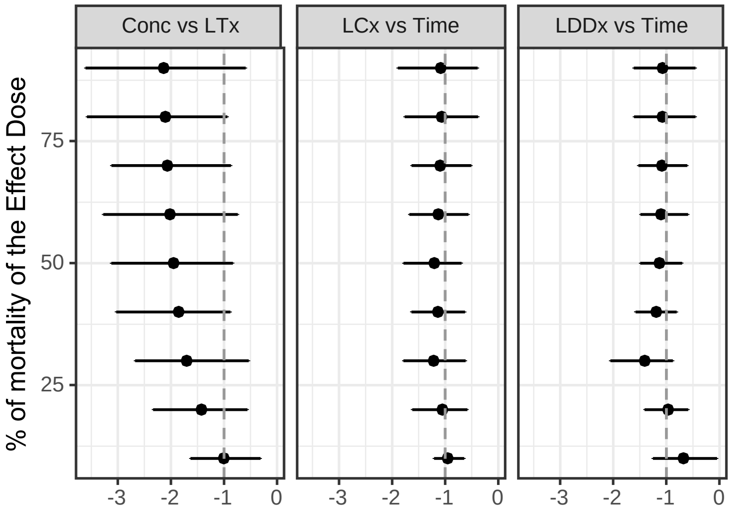

Supplementary Figure 1. Time-reinforced toxicity of FPF. Each dot represents the fixed effect slope of a random slope mixed model and the error bars are 95% bootstrap Confidence Intervals. Because the slopes of all toxicity endpoints (concentration, LCx, and LDDx) are not significantly different than what should be expected under Haber's rule (dashed grey line at −1), FPF toxicity is not time-reinforced.

Supplementary Table 1. Information on the seven participating laboratories, including the LT_{50} (Lethal Time for 50% of bees) of control treatment. The inter-laboratory performance of this ring test was satisfactory (z-score < 2; Lab #1: 1.2; #2: 0.0; #3: 1.7; #4: 0.7; #6: 1.2; #7: 0.2; only laboratories that reached the LT_{50} were included). Because laboratory #4 used 15 bees per cage instead of 20, it was excluded from the most sensitive sublethal assessments (food consumption, abnormal behaviours; see main text and SI annex for more details; $n_{survival}$ = 2494, $n_{sublethal}$ = 2222). The LT_{50} of laboratory #5 was not met by day 17 when their data were censored (technical issues, control mortality at day 17: 0%).

**Lab ID**	1	2	3	4	5	6	7
**Country**	Austria	France	Germany	Italy	Italy	Switzerland	USA
**Institution name**	University of Graz	Testapi	LLH - Bee Institute	Biotecnologie BT S.r.l.	Edmund Mach Foundation	University of Bern	University of California, San Diego
**Institution type**	Academia	Contract lab	Academia	Contract lab	Private Research Institution	Academia	Academia
**Coordinator**	Javier Hernandez-Lopez	Harve Giffard	Annely Brandt	Monica Colli	Valeria Malagnini	Geoffrey Williams	Simone Tosi
**Bee subspecies**	*A. m. carnica*	*A. m. Buckfast*	*A. m. carnica*	*A. m. ligustica*	*A. m. ligustica*	*A. m. carnica*	*A. m. ligustica*
**Colonies tested (N)**	3	3	3	3	3	3	3
**Bees per cage (N)**	20	20	20	15	20	20	20
**Bee age**	Newly emerged	Newly emerged	Newly emerged	Newly emerged	Newly emerged	Newly emerged	Newly emerged
**Anesthetization method**	None	None	None	None	None	None	None
**Trial T (°C)**	33 ± 2	33 ± 2	33 ± 2	33 ± 2	33 ± 2	33 ± 2	33 ± 2
**Trial RH (%)**	50 – 70	50 – 70	50 – 70	50 – 70	50 – 70	50 – 70	50 – 70
**Feeding diet**	50% (w/v) sucrose water, *ad lib*.	50% (w/v) sucrose water, *ad lib*.	50% (w/v) sucrose water, *ad lib*.	50% (w/v) sucrose water, *ad lib*.	50% (w/v) sucrose water, *ad lib*.	50% (w/v) sucrose water, *ad lib*.	50% (w/v) sucrose water, *ad lib*.
**Blind assessment**	No	No	No	No	No	No	Yes
**LT_{50} of control (day)**	20	26	34	24	>17	21	24

Supplementary Table 2. Main effects of FPF treatment on bee survival assessed over short-term (10 days[10]) or long-term (31 ± 5 days, complete experiment) exposure. We included the influence of colony and laboratory in the model (Fit Proportional Hazards) and report significant effects in bold.

**Incubation period (days)**	**Factor**	**DF**	**L-R χ^2**	***P*-value**
10	FPF treatment	5	343.92	**<0.0001**
	Colony	2	0.92	0.6311
	Laboratory	6	132.46	**<0.0001**
31 ± 5	FPF treatment	5	736.04	**<0.0001**
	Colony	2	12.34	**0.0021**
	Laboratory	6	368.92	**<0.0001**

Supplementary Table 3. Effects of FPF daily doses on bee survival assessed over short-term only (10 days[10]) or long-term (31 ± 5 days, complete experiment) exposure. The effect of each FPF treatment is compared with the control treatment. We report the Risk Ratios, which indicate the effect size (e.g., in the first row, RR of 1.7 corresponds to a 1.7 mortality increase caused by 11.1 ± 0.3 ng/bee/day (mean ± Standard Error of the Mean, SEM) as compared to control). We report significant effects in bold (Kaplan-Meier[DS]).

**Trial duration (days)**	**$FPF_{Daily\ dose}$ (ng/bee/day) (mean ± SEM)**	**χ^2**	**DF**	***P*-value**	**Risk Ratio**	***P*-value**
10	11.1 ± 0.3	3.82	1	0.0507	1.7	0.0350
	33.2 ± 0.7	0.46	1	0.4991	0.8	0.5983
	100.6 ± 2.2	0.11	1	0.7359	0.9	0.8425
	292.5 ± 8.1	0.36	1	0.5503	1.2	0.4584
	730.5 ± 28.4	169.60	1	**<0.0001**	11.1	**<0.0001**
31 ± 5	11.1 ± 0.3	6.09	1	**0.0136**	1.3	**0.0084**
	33.2 ± 0.7	0.84	1	0.3589	1.1	0.1384
	100.6 ± 2.2	10.70	1	**0.0011**	1.4	**0.0003**
	292.5 ± 8.1	44.13	1	**<0.0001**	1.9	**<0.0001**
	730.5 ± 28.4	514.92	1	**<0.0001**	9.3	**<0.0001**

Supplementary Table 4. Lethal Time (LT) 25, 50, and 75 (time until death of 25%, 50%, or 75% of bees, respectively) depending on the FPF daily dose received (reported as mean ± Standard Error of the Mean, SEM), assessed over short-term (10 days[10]) or long-term (31 ± 5 days, complete experiment) exposure. LTs were often not reached within 10 days ("NA").

Trial duration (days)	$FPF_{Daily\ dose}$ (ng/bee/day) (mean ± SEM)	LT_{25}	LT_{50}	LT_{75}
10	0	NA	NA	NA
	11.1 ± 0.3	NA	NA	NA
	33.2 ± 0.7	NA	NA	NA
	100.6 ± 2.2	NA	NA	NA
	292.5 ± 8.1	NA	NA	NA
	730.5 ± 28.4	9	NA	NA
31 ± 5	0	20	27	34
	11.1 ± 0.3	19	26	33
	33.2 ± 0.7	20	27	31
	100.6 ± 2.2	20	25	31
	292.5 ± 8.1	18	23	28
	730.5 ± 28.4	9	12	16

Supplementary Table 5. Main effects of FPF treatment on daily sucrose solution consumption per bee (mg/bee/24h) assessed for each 10 days of incubation to allow comparison between the standard 10 day chronic test[10] and longer-term exposures. We report in bold the significant effects (GLM).

Time range (days)	Factor	DF	L-R ChiSquare	*P*-value
1–10	FPF treatment	5	12.65	**0.0269**
1–10	Laboratory	5	27.50	**<0.0001**
1–10	Colony	2	0.99	0.6087
11–20	FPF treatment	5	30.23	**<0.0001**
11–20	Laboratory	5	37.45	**<0.0001**
11–20	Colony	2	0.11	0.9467
21–30	FPF treatment	5	17.05	**0.0044**
21–30	Laboratory	3	13.39	**0.0039**
21–30	Colony	2	2.58	0.2757
31–40	FPF treatment	5	11.23	**0.0470**
31–40	Laboratory	1	23.46	**<0.0001**
31–40	Colony	2	7.28	**0.0263**

Supplementary Table 6. Effects of dose on daily sucrose solution consumption per bee (mg per bee per day) assessed each 10 days of incubation, allowing comparison between the standard 10 day chronic test[10] and longer-term exposures. The effect of each FPF treatment is compared with the control treatment. We only tested comparisons with control based on visual estimation and report in bold significant effects after Dunn-Sidak correction (contrast test[DS]). We tested specific dose effects only when the main treatment effect was significant (GLM, Supplementary Table 4).

Time range (days)	$FPF_{Daily\ dose}$ (ng/bee/day)	DF	L-R ChiSquare	*P*-value
1–10	11.1 ± 0.3	1	0.02	0.8910
	33.2 ± 0.7	1	0.02	0.9002
	100.6 ± 2.2	1	0.07	0.7972
	292.5 ± 8.1	1	0.06	0.7999
	730.5 ± 28.4	1	8.07	**0.0045**
11–20	11.1 ± 0.3	1	0.69	0.4046
	33.2 ± 0.7	1	2.51	0.1130
	100.6 ± 2.2	1	0.11	0.7384
	292.5 ± 8.1	1	1.74	0.1874
	730.5 ± 28.4	1	25.20	**<0.0001**
21–30	11.1 ± 0.3	1	0.21	0.6452
	33.2 ± 0.7	1	7.44	**0.0064**
	100.6 ± 2.2	1	8.10	**0.0044**
	292.5 ± 8.1	1	2.70	0.1004
	730.5 ± 28.4	1	8.75	**0.0031**
31–40	11.1 ± 0.3	1	3.40	0.0652
	33.2 ± 0.7	1	0.04	0.8432
	100.6 ± 2.2	1	2.70	0.1003
	292.5 ± 8.1	1	6.80	**0.0091**
	730.5 ± 28.4	1	0.21	0.6490

Supplementary Table 7. Summary of effect size measures representing the decrease in food consumption after exposure to each FPF treatment across time. Food consumption weight per FPF treatment (reported as mean ± Standard Error of the Mean, SEM) and time category was compared to the respective control treatment per each time category. The results are reported as percentage change to describe relatively smaller effect sizes more accurately, as compared to abnormal behaviour effects.

	Time (days after treatment)			
	**1–10**	**11–20**	**21–30**	**31–40**
**$FPF_{Daily\ dose}$ (ng/bee/day) (mean ± SEM)**	**Effect size measures**			
**11.1 ± 0.3**	1	4	5	24
**33.2 ± 0.7**	1	13	13	–5
**100.6 ± 2.2**	1	4	18	29
**292.5 ± 8.1**	3	9	19	28
**730.5 ± 28.4**	16	28	34	59

Supplementary Table 8. Daily dose of sucrose solution (zero FPF concentration) and FPF consumed by bees depending on the FPF concentration administered during incubation. Daily doses are based upon consumption, and thus provide more accurate information as compared to concentration in terms of pesticide intake. Consumption values are defined taking in consideration the evaporation rate per laboratory per day and the number of alive bees per cage per day. We report the mean and the Standard Error of the Mean (SEM).

FPF concentration (µg/kg)	Sucrose solution daily consumption (mg/bee/24h)			FPF daily consumption (ng/bee/day)		
	**N**	**Mean**	**SEM**	**N**	**Mean**	**SEM**
0	563	26.5	0.5	563	0.0	0
444	481	25.5	0.5	482	11.1	0.3
1333	465	24.9	0.5	465	33.2	0.7
4000	459	25.2	0.5	460	100.6	2.2
12000	450	24.4	0.7	451	292.5	8.1
36000	347	20.6	0.8	353	730.5	28.4

Supplementary Table 9. Daily dose (reported as mean and Standard Error of the Mean, SEM) of 50% sucrose solution consumed by bees. The results are reported for both control bees (pesticide-free) and those exposed to FPF. We display the results in relation to age of the bee (by 10-day time blocks). Results of pure sucrose solution (containing a FPF concentration of zero) represent a baseline for honey bee consumption over most of the organism lifespan. Consumption values are defined taking in consideration the evaporation rate per laboratory per day and the number of alive bees per cage per day. We do not show data after 40 days of age given the corresponding limited bee survival.

FPF concentration (μg/kg)	Bee age (days interval)	Daily sucrose solution consumption (mg/bee/24h)		
		N	Mean	SEM
0	1–10	210	26.0	0.7
	11–20	197	28.2	0.7
	21–30	105	27.8	1.5
	31–40	38	23.6	2.8
444	1–10	180	25.8	0.7
	11–20	168	26.9	0.7
	21–30	87	26.5	1.2
	31–40	44	17.9	2.4
1333	1–10	180	25.6	0.8
	11–20	168	24.6	0.9
	21–30	87	24.2	0.9
	31–40	30	24.7	3.7
4000	1–10	180	25.9	0.7
	11–20	167	27.0	0.8
	21–30	87	22.8	1.5
	31–40	25	16.9	3.2
12000	1–10	180	25.1	1.2
	11–20	161	25.7	0.9
	21–30	86	22.6	1.5
	31–40	23	17.0	3.3
36000	1–10	180	21.8	0.8
	11–20	123	20.3	1.6
	21–30	36	18.5	3.0
	31–40	8	9.7	4.8

Supplementary Table 10. Main effects of FPF treatment on the proportion of living bees exhibiting abnormal behaviours per cage per day. The data were grouped each 10 days of incubation, allowing comparison between the standard 10 day chronic test[10] and longer-term exposures. We report in bold the significant effects (GLM).

Time range (days)	Factor	DF	L-R ChiSquare	*P*-value
1–10	FPF treatment	5	139.94	**<0.0001**
1–10	Laboratory	5	90.59	**<0.0001**
1–10	Colony	2	0.81	0.6669
11–20	FPF treatment	5	134.40	**<0.0001**
11–20	Laboratory	5	61.40	**<0.0001**
11–20	Colony	2	1.28	0.5272
21–30	FPF treatment	5	18.06	**0.0029**
21–30	Laboratory	3	35.06	**<0.0001**
21–30	Colony	2	0.49	0.7841
31–40	FPF treatment	5	9.01	0.1087
31–40	Laboratory	1	5.28	**0.0216**
31–40	Colony	2	2.52	0.2830

Supplementary Table 11. Effects of dose on the proportion of living bees exhibiting abnormal behaviours per cage per day. The data were grouped each 10 days of incubation, allowing comparison between the standard 10 day chronic test[10] and longer-term exposures. We tested specific dose effects only when the main treatment effect was significant (GLM, Supplementary Table 8). Based upon visual inspection of the data, we performed limited comparisons with the control treatment and report in bold effects that remained significant after Dunn-Sidak correction (contrast test[DS]).

**Time range (days)**	**$FPF_{Daily\ dose}$ (ng/bee/day)**	**DF**	**L-R ChiSquare**	***P*-value**
1–10	11.1 ± 0.3	1	12.16	**0.0005**
1–10	33.2 ± 0.7	1	16.10	**0.0001**
1–10	100.6 ± 2.2	1	52.93	**<0.0001**
1–10	292.5 ± 8.1	1	16.52	**<0.0001**
1–10	730.5 ± 28.4	1	96.43	**<0.0001**
11–20	11.1 ± 0.3	1	9.16	**0.0025**
11–20	33.2 ± 0.7	1	11.78	**0.0006**
11–20	100.6 ± 2.2	1	10.64	**0.0011**
11–20	292.5 ± 8.1	1	14.74	**0.0001**
11–20	730.5 ± 28.4	1	16.84	**<0.0001**
21–30	11.1 ± 0.3	1	1.89	0.1689
21–30	33.2 ± 0.7	1	1.33	0.2490
21–30	100.6 ± 2.2	1	2.08	0.1495
21–30	292.5 ± 8.1	1	6.44	**0.0111**
21–30	730.5 ± 28.4	1	6.75	**0.0094**

Supplementary Table 12. Summary of effect size measures representing the increase of bees exhibiting at least an abnormal behaviour after exposure to each FPF treatment across time. Each proportion of abnormally behaving bees per FPF treatment per time category was compared to the respective control treatment per each time category via fold-change or percentage-change methods (i.e. in the first ten days after treatment, the lowest FPF dose caused a 6-fold (460%) increment in the proportion of bees exhibiting abnormal behaviours).

Method	$FPF_{Daily\ dose}$ (ng/bee/day) (mean ± SEM)	Time (days after treatment) 1–10	11–20	21–30	31–40
		Effect size measures			
Fold-change	11.1 ± 0.3	6	4	2	2
	33.2 ± 0.7	20	5	2	2
	100.6 ± 2.2	18	4	2	4
	292.5 ± 8.1	28	10	4	3
	730.5 ± 28.4	158	44	4	6
Percentage change	11.1 ± 0.3	460	253	56	93
	33.2 ± 0.7	1860	410	61	73
	100.6 ± 2.2	1675	328	83	311
	292.5 ± 8.1	2714	873	318	189
	730.5 ± 28.4	15711	4305	347	532

Supplementary Table 13. FPF time-reinforced results of the fixed effects slope of a random slope mixed model and 95% bootstrap Confidence Intervals. We tested each Effective Dose (ED), including the Lethal Time (LT), the Lethal Concentration (LC), and the Lethal Daily Dose (LDD), at multiple effect points (10–90% mortality). Further information is available in the SI methods, Fig. S1, and the Figshare public repository.

Method	EDx	Intercept	Slope	CIlower	CIupper	Haber_int
Conc vs LTx	LT10	3.841	−1.002	−1.631	−0.3126	3.836
Conc vs LTx	LT20	5.299	−1.423	−2.335	−0.5589	4.141
Conc vs LTx	LT30	6.294	−1.702	−2.674	−0.5327	4.291
Conc vs LTx	LT40	6.919	−1.854	−3.031	−0.8800	4.396
Conc vs LTx	LT50	7.368	−1.950	−3.124	−0.8311	4.482
Conc vs LTx	LT60	7.728	−2.016	−3.275	−0.7405	4.560
Conc vs LTx	LT70	8.045	−2.065	−3.121	−0.8685	4.638
Conc vs LTx	LT80	8.355	−2.104	−3.581	−0.9369	4.725
Conc vs LTx	LT90	8.710	−2.134	−3.609	−0.5901	4.842
LCx vs Time	LC10	5.245	−0.951	−1.211	−0.6424	5.376
LCx vs Time	LC20	5.677	−1.051	−1.621	−0.5834	5.538
LCx vs Time	LC30	6.325	−1.216	−1.786	−0.6138	5.723
LCx vs Time	LC40	6.266	−1.137	−1.636	−0.6265	5.887
LCx vs Time	LC50	6.544	−1.206	−1.784	−0.6911	5.971
LCx vs Time	LC60	6.432	−1.128	−1.674	−0.5623	6.071
LCx vs Time	LC70	6.427	−1.093	−1.633	−0.5085	6.159
LCx vs Time	LC80	6.447	−1.067	−1.763	−0.3810	6.252
LCx vs Time	LC90	6.610	−1.084	−1.892	−0.3883	6.361
LDDx vs Time	LDD10	0.630	−0.678	−1.255	−0.0466	1.510
LDDx vs Time	LDD20	1.652	−0.967	−1.410	−0.5889	1.742
LDDx vs Time	LDD30	2.983	−1.407	−2.056	−0.8767	1.895
LDDx vs Time	LDD40	2.584	−1.190	−1.581	−0.8057	2.087
LDDx vs Time	LDD50	2.549	−1.131	−1.492	−0.7025	2.200
LDDx vs Time	LDD60	2.565	−1.099	−1.487	−0.5906	2.293
LDDx vs Time	LDD70	2.615	−1.082	−1.529	−0.6072	2.382
LDDx vs Time	LDD80	2.693	−1.074	−1.609	−0.4513	2.477
LDDx vs Time	LDD90	2.817	−1.072	−1.616	−0.4513	2.601

Supplementary Table 14. Comparison of the key method refinements used in our experiment, as compared to the OECD official ecotoxicological guideline[10]. Our ring test experiment (including 7 valid laboratory trials) lasted 31 ± 5 days (mean ± SE).

**Parameters**	**OECD, 2017**	**Tosi et al.**
Individuals per replicate (N)	10	20
Colonies used (N)	1	3
Trial duration (days)	10	31 ± 5 days (mean ± SE)<br>(at least LT_{50} of control treatment)

Supplementary Table 15. Food evaporation rate by each laboratory involved in the food consumption assessment. Each laboratory measured evaporation (as sucrose solution weight) from three cages, which were maintained at the same conditions as all cages but did not contain bees. The measurements occurred daily and were used to assess the daily consumption of sucrose and pesticide by bees. The daily average evaporation rate was calculated considering the total weight of food administered per cage per day and its respective weight loss after 24h of evaporation. The overall evaporation rate is calculated as the average of each individual lab. Evaporation data from each laboratory were used to correct food consumption estimations for the same laboratory.

**Lab ID**	**N**	**Average daily evaporation rate (%)**
1	60	0.8
2	78	2.3
3	108	0.9
4	72	1.8
5	51	2.1
6	69	0.6
7	159	1.9
Overall	*597*	*1.5*

Supplementary References

1. Campbell, J. W., Cabrera, A. R., Stanley-Stahr, C. & Ellis, J. D. An evaluation of the honey bee (Hymenoptera: Apidae) safety profile of a new systemic insecticide, flupyradifurone, under field conditions in Florida. *J. Econ. Entomol.* **96**, 875–878 (2016).
2. US EPA. *Environmental fate and ecological risk assessment for foliar, soil drench, and seed treatment uses of the new insecticide flupyradifurone (BYI 02960)*. (2014).
3. EFSA. EFSA Guidance Document on the risk assessment of plant protection products on bees (Apis mellifera, Bombus spp. and solitary bees) - version 2014. *EFSA J.* **11**, 268 (2013).
4. Crane, E. *Honey. A comprehensive survey*. (Heinemann, 1975).
5. Pierre, J., Mesquida, J., Marilleau, R., Pham-Delègue, M. H. & Renard, M. Nectar secretion in winter oilseed rape, Brassica napus - Quantitative and qualitative variability among 71 genotypes. *Plant Breed.* **118**, 471–476 (1999).
6. Rortais, A. *et al.* Risk assessment of pesticides and other stressors in bees: Principles, data gaps and perspectives from the European Food Safety Authority. *Sci. Total Environ.* **587**–**588**, 524–537 (2017).
7. US EPA. *White paper in support of the proposed risk assessment process for bees*. (2012).
8. Bates, D., Mächler, M., Bolker, B. M. & Walker, S. C. Fitting linear mixed-effects models using lme4. *J. Stat. Softw.* **67**, (2015).
9. Ritz, C., Baty, F., Streibig, J. C. & Gerhard, D. Dose-response analysis using R. *PLoS One* **10**, 1–13 (2015).
10. OECD/OCDE. *OECD guideline 245 for the testing of chemicals. Honey bee (Apis mellifera L.), chronic oral toxicity test (10-day feeding)*. *OECD/OCDE* (OECD Publishing, 2017). doi:10.1787/9789264284081-en
11. Tosi, S. & Nieh, J. C. Lethal and sublethal synergistic effects of a new systemic pesticide, flupyradifurone (Sivanto®) on honey bees. *Proc. R. Soc. B Biol. Sci.* **286**, 20190433 (2019).
