# Supplementary material for: Long-term field-realistic exposure to a next-generation pesticide, flupyradifurone, impairs honey bee behaviour and survival
Source: Commun Biol. 2021 Jun 28;4:805. doi: 10.1038/s42003-021-02336-2 (PMC8238954; doi:10.1038/s42003-021-02336-2)
Supplement: Supplementary file 2 — Reporting Summary [file 42003_2021_2336_MOESM2_ESM.pdf]

nature research

Corresponding author(s): Simone Tosi

Last updated by author(s): Apr 19, 2021

Reporting Summary

Nature Research wishes to improve the reproducibility of the work that we publish. This form provides structure for consistency and transparency in reporting. For further information on Nature Research policies, see our Editorial Policies and the Editorial Policy Checklist.

Statistics

For all statistical analyses, confirm that the following items are present in the figure legend, table legend, main text, or Methods section.

n/a	Confirmed	
☐	☒	The exact sample size (n) for each experimental group/condition, given as a discrete number and unit of measurement
☐	☒	A statement on whether measurements were taken from distinct samples or whether the same sample was measured repeatedly
☐	☒	The statistical test(s) used AND whether they are one- or two-sided<br>*Only common tests should be described solely by name; describe more complex techniques in the Methods section.*
☐	☒	A description of all covariates tested
☐	☒	A description of any assumptions or corrections, such as tests of normality and adjustment for multiple comparisons
☐	☒	A full description of the statistical parameters including central tendency (e.g. means) or other basic estimates (e.g. regression coefficient) AND variation (e.g. standard deviation) or associated estimates of uncertainty (e.g. confidence intervals)
☐	☒	For null hypothesis testing, the test statistic (e.g. F, t, r) with confidence intervals, effect sizes, degrees of freedom and P value noted<br>*Give P values as exact values whenever suitable.*
☐	☒	For Bayesian analysis, information on the choice of priors and Markov chain Monte Carlo settings
☐	☒	For hierarchical and complex designs, identification of the appropriate level for tests and full reporting of outcomes
☐	☒	Estimates of effect sizes (e.g. Cohen's d, Pearson's r), indicating how they were calculated

Our web collection on statistics for biologists contains articles on many of the points above.

Software and code

Policy information about availability of computer code

Data collection	No software was used to collect data.
Data analysis	We used JMP v14.0.0 (SAS Statistical Software) and R Studio (R Core Team. R: a language and environment for statistical computing, 2016) software to analyse the data. The codes used are provided.

For manuscripts utilizing custom algorithms or software that are central to the research but not yet described in published literature, software must be made available to editors and reviewers. We strongly encourage code deposition in a community repository (e.g. GitHub). See the Nature Research guidelines for submitting code & software for further information.

Data

Policy information about availability of data

All manuscripts must include a data availability statement. This statement should provide the following information, where applicable:

- Accession codes, unique identifiers, or web links for publicly available datasets
- A list of figures that have associated raw data
- A description of any restrictions on data availability

The survival and behavioural data, as well as the R script, are available in the SI or the open access data repository Figshare.

Field-specific reporting

Life sciences study design

All studies must disclose on these points even when the disclosure is negative.

Sample size	Sample size was determined based on official and standard experimental guidelines from OECD and EFSA. Our sample size was higher than official and standard guidelines.<br><br>OECD/OCDE. OECD guideline 245 for the testing of chemicals. Honey bee (Apis mellifera L.), chronic oral toxicity test (10-day feeding). OECD/OCDE (OECD Publishing, 2017). doi:10.1787/9789264284081-en<br>OECD/OCDE. OECD Guideline 213 for the testing of chemicals on honeybee, acute oral toxicity test. (1998). doi:10.1787/9789264070165-en<br>EFSA. EFSA Guidance Document on the risk assessment of plant protection products on bees (Apis mellifera, Bombus spp. and solitary bees) - version 2014. EFSA J. 11, 268 (2013).
Data exclusions	All details about data exclusion are reported in the manuscript, main text (Methods section) and SI. Here is a summary:<br>As mandatory in all risk assessment trials, exclusion criteria are based on pre-established validity criteria. Our experiment complies, as we used the standard official pre-established validity criteria (EFSA, OECD).<br>Among the seven laboratories that participated to the experiment (Supplementary Table 1), one (Lab #4) was excluded from the sublethal (food consumption, abnormal behaviours) assessments because 15 bees per cage instead of 20 were used.<br>The LT50 of Lab #5 was not met by day 17, when their data were censored (Supplementary Table 1, technical issues). Thus, Lab #5 could not be used in the inter-laboratory assessment as it required reaching the LT50.<br><br>OECD/OCDE. OECD guideline 245 for the testing of chemicals. Honey bee (Apis mellifera L.), chronic oral toxicity test (10-day feeding). OECD/OCDE (OECD Publishing, 2017). doi:10.1787/9789264284081-en<br>OECD/OCDE. OECD Guideline 213 for the testing of chemicals on honeybee, acute oral toxicity test. (1998). doi:10.1787/9789264070165-en<br>EFSA. EFSA Guidance Document on the risk assessment of plant protection products on bees (Apis mellifera, Bombus spp. and solitary bees) - version 2014. EFSA J. 11, 268 (2013).
Replication	The experiment was replicated, following the same main protocol, in multiple laboratories (EU and USA). As expected and usual in honey bee ecotoxicological research, the results can be influenced by multiple environmental and biological factors (e.g. it is known that even the reference toxicological value adopted globally, the LD50, can vary when tests are replicated). This is complicated by the intrinsic complexity of the superorganism honey bee. See EFSA Guidance (2013) as example.<br>EFSA. EFSA Guidance Document on the risk assessment of plant protection products on bees (Apis mellifera, Bombus spp. and solitary bees) - version 2014. EFSA J. 11, 268 (2013).
Randomization	All organisms were randomly allocated in different cages. Cages were randomly assigned to each treatment group.
Blinding	Blinding was not performed in this study given the complexity of the experimental design. The laboratories participating are used to these types of tests and aware of the importance of objective observations.

Reporting for specific materials, systems and methods

We require information from authors about some types of materials, experimental systems and methods used in many studies. Here, indicate whether each material, system or method listed is relevant to your study. If you are not sure if a list item applies to your research, read the appropriate section before selecting a response.

Materials & experimental systems

n/a	Involved in the study	
☒	☐	Antibodies
☒	☐	Eukaryotic cell lines
☒	☐	Palaeontology and archaeology
☐	☒	Animals and other organisms
☒	☐	Human research participants
☒	☐	Clinical data
☒	☐	Dual use research of concern

Methods

n/a	Involved in the study	
☒	☐	ChIP-seq
☒	☐	Flow cytometry
☒	☐	MRI-based neuroimaging

Animals and other organisms

Policy information about studies involving animals; ARRIVE guidelines recommended for reporting animal research

Laboratory animals	The honey bee (Apis mellifera) was used. Newly-emerged worker females were specifically used in the lab experiments.
Wild animals	The study did not involve wild animals.
Field-collected samples	Honey bee colonies were used to extract the newly-emerged worker females. The honey bee colony is a superorganism that can be managed by beekeepers in experimental apiaries. They were kept healthy and monitored, following standard beekeeping and inspection techniques (see below). The honey bee colonies were maintained alive after the experiment, common practice in

beekeeping and honey bee research.

Dietemann, V. et al. Standard methods for varroa research. J. Apic. Res. 52, 1–54 (2013).
Fries, I. et al. Standard methods for nosema research. J. Apic. Res. 52, 1–28 (2013).

Ethics oversight	No ethical approval was required as we only tested the honey bee.

Note that full information on the approval of the study protocol must also be provided in the manuscript.
